# Sequence and Structure Analysis of Biological Molecules Based on Computational Methods

**DOI:** 10.1155/2015/565328

**Published:** 2015-07-05

**Authors:** Jia-Feng Yu, Yue-Dong Yang, Xiao Sun, Ji-Hua Wang

**Affiliations:** ^1^Shandong Provincial Key Laboratory of Functional Macromolecular Biophysics, Institute of Biophysics, Dezhou University, Dezhou 253023, China; ^2^Institute for Glycomics, Griffith University, Southport, QLD 4222, Australia; ^3^State Key Laboratory of Bioelectronics, Southeast University, Nanjing 210096, China; ^4^College of Physics and Electronic Information, Dezhou University, Dezhou 253023, China

The number of sequences and structures of biological molecules, such as DNA, RNA, and proteins, is rapidly increasing in databases. According to the statistics in the newest version of GOLD database (https://gold.jgi-psf.org/) [1], 6651 genomes have been completed and published and 51954 genomes are of permanent drafts or incomplete. The protein data bank (PDB, http://www.rcsb.org/) [2] has published 107958 biological macromolecular structures, including 35542 protein sequences, 28142 structures of human sequences, and 7611 nucleic acid containing structures. To discover the underlying mechanism behind the information, it is necessary to develop effective feature parameters for representing the sequence and structure. Although many studies have been proposed for sequence and structure analysis, more and more studies indicated that the problem is far from solved [3, 4]. How to computationally analyze the big data of biologically molecular sequences and structures has been one of the major challenges in bioinformatics. Machine learning and optimization methods are important methods for the analyses. This special issue focuses on recent progress of the computational methods for biological sequences or structures studies. Correspondingly, after a rigorous peer review, eleven papers were selected. We briefly describe these papers as follows.

In “Predicting Homogeneous Pilus Structure from Monomeric Data and Sparse Constraints,” K. Xiao et al. developed a new approach to predict pseudoatomic models of* pili* by combining ambiguous symmetric constraints with sparse distance information obtained from experiments. The method was successfully implemented to the reconstruct the gonococcal (GC) pilus from* Neisseria gonorrhoeae*. A global sampling in a wide range implied that a pilus might have more than one but fewer than many possible intact conformations.

In “Evolutionary and Expression Analysis of miR-#-5p and miR-#-3p at the miRNAs/isomiRs Levels,” L. Guo et al. explored the potential evolutionary and expression divergence and relationships between miRNAs from different arms of different/same pre-miRNAs according to the arm selection and/or arm switching phenomenon in miRNA world. They found no bias in the numbers but different nucleotide compositions between 5p-miRNA and 3p-miRNA. IsomiR expression profiles from the two arms are always stable, but isomiR expressions in diseased samples are prone to show larger degree of dispersion. miR-#-5p and miR-#-3p have relative independent evolution/expression patterns and datasets of target mRNAs, which might also contribute to the phenomena of arm selection and/or arm switching. Simultaneously, miRNA/isomiR expression profiles may be regulated via arm selection and/or arm switching, and the dynamic miRNAome and isomiRome will adapt to functional and/or evolutionary pressures. A comprehensive analysis and further experimental study at the miRNA/isomiR levels are quite necessary for miRNA study.

In “Strong Ligand-Protein Interactions Derived from Diffuse Ligand Interactions with Loose Binding Sites,” L. Marsh presented a fine-grained computational method for numerical integration of total binding free energy arising from diffuse regional interaction of a ligand in multiple conformations using a Markov Chain Monte Carlo. The application to the bacterial multidrug efflux pump AcrB indicated that diffuse binding effects could cause 100-fold binding affinity for some ligands while little role in the binding of other ligands. Analysis of other proteins with large binding pockets indicated that the influence of this process varies greatly, dependent on ligand and protein target. This work may be of broad interest to those studying a variety of biological systems including protein folding, DNA-protein binding, and drug-receptor docking that depend on dispersed interactions.

In “Redesigning Protein Cavities as a Strategy for Increasing Affinity in Protein-Protein Interaction: Interferon-*γ* Receptor 1 as a Model,” J. Černý et al. presented a new strategy for designing high affinity variants of a binding protein through mutating residues at positions lining internal cavities of one of the interacting molecules instead of at the interface. The test on interferon-*γ* receptor 1 has brought up to sevenfold increase in the binding affinity. Analysis shows that the affinity increase is linked to the restriction of molecular fluctuations in the unbound state of the receptor. This serves as an example of a viable strategy for designing protein variants with increased affinity.

In “Multi-Instance Multilabel Learning with Weak-Label for Predicting Protein Function in Electricigens,” J.-S. Wu et al. have applied the state-of-the-art MIML with weak-label learning algorithm MIMLwel for predicting protein functions in two typical real-world electricigens organisms widely used in microbial fuel cells studies. The experimental results validate the effectiveness of MIMLwel algorithm in predicting protein functions with incomplete annotation.

In “Detecting Protein-Protein Interactions with a Novel Matrix-Based Protein Sequence Representation and Support Vector Machines,” Z.-H. You et al. developed a novel computational approach to effectively detect the protein interactions based on a novel matrix-based representation of protein sequence by SVM. The sequence information includes the order of amino acids and composition of dipeptide. The prediction was proven highly accurate on the benchmark of yeast PPIs datasets. Thus, it can be a helpful supplement to the missing data and false positive by experimental results from high-throughput techniques.

In “An Improved Method for Completely Uncertain Biological Network Alignment,” B. Shen et al. designed an improved method to analyze uncertain biological networks by complete probabilistic biological network alignment. This method has solved the weakness of PBNA by allowing both networks to be probabilistic; thus, it could take full advantage of the uncertain information of biological network. The new method was proven to consistently improve over PBNA in both GO Consistency and Global Network Alignment Score.

In “A Systematic Analysis of Candidate Genes Associated with Nicotine Addiction,” M. Liu et al. have performed a systematic analysis on a set of nicotine addiction-related genes (NAGenes) to explore their characteristics at network levels. They found that NAGenes tended to have a more moderate degree, weaker clustering coefficient in the network, and are less central in the network compared to genes related to alcohol addiction or cancer. In addition, an intuitional view was provided to understand their major molecular functions by six clusters from the clustering of these genes with themes in synaptic transmission, signal transduction, metabolic process, and apoptosis. Moreover, it was found that nicotine addiction involves neurodevelopment, neurotransmission activity, and metabolism related biological functions. The systematic network and functional enrichment analysis for nicotine addiction in this study is valuable for understanding the molecular mechanisms underlying nicotine addiction.

In “Constraint Programming based Biomarker Optimization,” M. Zhou et al. proposed an algorithm for highly accurate feature selection for biomedical classification problem, while allowing the inclusion of user-input constraints for the optimization process. The experimental results showed that the proposed method provided flexibility of allowing both the well-known disease biomarkers like P53 and the existing feature selection algorithms' results as the constraints, while achieving promising classification performances. This work provided useful tool for efficiently exploring the health big data, consisting of both the bio-OMIC data and huge amount of knowledge accumulated in the literature and other sources.

In “An Improved Opposition-Based Learning Particle Swarm Optimization for the Detection of SNP-SNP Interactions,” J. Shang et al. presented an improved method for detecting SNP-SNP interactions by using opposition-based learning particle swarm optimization. The algorithm has ensured the ability of global searching and prevented premature convergence. The application to a dataset of age-related macular degeneration shows the strength of the method on real applications by capturing important features of genetic architecture not previously discovered. The method provides an important tool in detecting SNP-SNP interactions in future.

In “Nucleosome Organization around Pseudogenes in the Human Genome,” G. Liu et al. investigated the effect of nucleosome positioning on pseudogene transcription. The results show that, for transcribed pseudogenes, nucleosomes upstream of the start positions and end positions of transcribed pseudogenes are depleted. Interestingly, the same depletion is also observed for nontranscribed pseudogenes. The consistent pattern of sequence-dependent prediction with the assessment by experimental data indicates that sequence-dependent mechanism of nucleosome positioning may play important roles in both the transcription initiation and termination of pseudogenes.
